# Self-reported use of cannabidiol as a substitute or adjunct for approved medications

**DOI:** 10.3389/fpubh.2026.1720348

**Published:** 2026-02-06

**Authors:** Emily A. C. Austin, Lara Berghammer, Shannon E. Ellis, Giovanni Appolon, Jenna Brooks, Nina M. Rice, Prosperity Land, Siyuan Ping, Nora Satybaldiyeva, Igor Grant, Eric C. Leas

**Affiliations:** 1 Herbert Wertheim School of Public Health and Human Longevity Science, University of California San Diego, La Jolla, CA, United States; 2 Department of Cognitive Science, University of California San Diego, La Jolla, CA, United States; 3 Halıcıŏglu Data Science Institute, University of California San Diego, La Jolla, CA, United States; 4 Department of Political Science, University of California San Diego, La Jolla, CA, United States; 5 Stanford University School of Medicine, Stanford, CA, United States; 6 Center for Medicinal Cannabis Research, Department of Psychiatry, University of California San Diego, La Jolla, CA, United States

**Keywords:** cannabidiol, drug interactions, drug–drug interactions, medical conditions, public health, substitute and adjunct use, substitute or adjunct therapy

## Abstract

**Objective:**

To assess the prevalence of CBD use among US adults, identify the health conditions for which CBD is used, and examine whether CBD is used as a substitute or adjunct to conventional treatments.

**Design, setting, and participants:**

This cross-sectional survey used Ipsos KnowledgePanel®, a US probability-based online panel covering approximately 97% of US adults and including households without internet access (which are provided internet service and/or devices). Between October 25 and November 3, 2023, a random sample of 4,505 US adults was invited; 2,880 responded (63.9% completion) and 1,523 qualified (1,008 ever CBD users and 515 never users). The survey was administered in English and Spanish and weighted analyses provided nationally representative estimates.

**Exposures:**

Self-reported use of CBD as a substitute (replacement) for or adjunct (combined use) with another medication.

**Main outcomes and measures:**

Respondents reporting substitute or adjunct use listed specific health conditions and medications; these were coded using the Medical Dictionary for Regulatory Activities (MedDRA) and RxNav, respectively.

**Results:**

An estimated 35.2% (95% CI, 32.7–37.9) of US adults (approximately 90.8 million) reported ever using CBD, and 21.8% (95% CI, 20.0–23.8) reported use in the past 12 months. Among ever CBD users, 32.0% (95% CI, 29.1–35.1) had used CBD as a substitute or adjunct for at least one medication. Adjunct use (24.2%; 95% CI, 21.5–27.1) was more common than substitute use (11.0%; 95% CI, 9.1–13.3). The conditions most frequently targeted were musculoskeletal and connective tissue disorders (e.g., joint pain; 10.1%; 95% CI, 8.4–12.2), psychiatric disorders (e.g., anxiety; 7.4%; 95% CI, 5.9–9.2), and general disorders or administration site conditions (e.g., procedural pain; 6.8%; 95% CI, 5.4–8.6). Frequently replaced or co-administered medications included ibuprofen (4.8, 95% CI: 3.6–6.3), Tylenol (3.9, 95% CI: 2.8–5.3), and other over-the-counter analgesics. Only a small proportion of CBD ever users reported ever having a health problem they believed resulted from CBD use (2.4%; 95% CI: 1.5–3.6).

**Conclusions and relevance:**

CBD use as a substitute or adjunct to medications was common among US adults particularly for pain medications. These patterns underscore the need for better evidence and clearer guidance on dosing, product quality, and co-use with other treatments.

## Introduction

Since the passage of the 2014 Farm Bill, which reduced federal restrictions on cannabis cultivation and manufacturing (1), the popularity of products containing the cannabis-derived compound cannabidiol (CBD) has grown considerably, with a market reaching consumers in all 50 US states (2). Increased consumer interest was spurred in part by the widespread marketing of CBD as a universal remedy (3, 4). However, currently, the US Food and Drug Administration (FDA) has not approved any CBD products other than one prescription drug (Epidiolex®), indicated for treating Lennox–Gastaut syndrome, Dravet syndrome, and, more recently, tuberous sclerosis complex (three rare pediatric epilepsy disorders) (5–7). Moreover, in 2023, the FDA also concluded that existing regulatory pathways available to manufacturers—including the pathway dietary supplement development—are “not appropriate for CBD,” citing various safety concerns including “the potential for harm to the liver, interactions with certain medications and possible harm to the male reproductive system” (8).

Although other therapeutic applications for CBD show promise (9), and broader investigation of CBD may increase as restrictions on medical cannabis research are relaxed (10), consumers may already be using CBD for indications that currently demonstrate no therapeutic benefit, thereby exposing themselves to potential side effects without meaningful clinical gain (11, 12). Recent observational studies suggest that individuals often turn to CBD to manage chronic pain, anxiety, and sleep disorders as a substitute or adjunct to FDA-approved treatments for these conditions and many of these clinical endpoints for CBD do show promise (13–16). For example, a recent review examining patient outcomes with the use of cannabis-based products for chronic pain found CBD to be effective in many cases (17). However, the potential benefits of CBD should be balanced with potential risks stemming from the current market, including several known side effects of CBD [e.g., male reproductive toxicity, nausea, vomiting, decreased appetite, sedation and somnolence (18, 19) and issues with product quality that could result in adverse events (20, 21)]. For a perspective on the prevalence of CBD adverse events, between 2020 and 2024 US poison control centers reported managing 11,402 cases related to CBD (22), which is more than three times the number of cases reported for synthetic cannabinoid products (*n* = 3,545), such as K2 and Spice, over the same time period despite widespread safety concerns about synthetic cannabinoid products (23).

To address the risk of unproven therapeutic applications or misuse of CBD, the FDA has issued several warning letters to manufacturers for illegally selling CBD products claiming to treat medical conditions (24, 25). In congressional testimony the FDA commissioner stated the agency would take stronger and wider-ranging actions than issuance of warnings if clear evidence emerged that patients with diagnosable conditions were using CBD as a substitute or adjunct for approved therapies (26). A delayed response by the FDA to take additional action against illegal selling and unapproved marketing of unsubstantiated health claims may in part be due to the lack of data available to answer simple epidemiological questions like, “What is the prevalence of CBD use?” and “Why do people use CBD?.” (27) Three nationally representative surveys have documented past-year CBD use among US adults at 14% in 2019 (28), 20.6% in 2022 (29), and 21.1% in June 2023 (30). However, existing observational research documenting reasons for use is limited to convenience samples or has not asked about using CBD as a substitute or adjunct to conventional treatments, which is critical for understanding the risks of consumers’ cessation of evidence-based therapies and specific drug–drug interactions when CBD is used as an adjunct. To better characterize how CBD is being integrated into current treatment regimens across a broad population, we conducted a nationally representative survey assessing individuals’ patterns of CBD use among US adults. We specifically examined whether respondents utilized CBD as a substitute or adjunct therapy for conventional medications, the most commonly targeted conditions, and the medications they substituted or combined with CBD. We also estimate past-year use to compare with existing nationally representative surveys.

## Methods

Funded by the US National Institute on Drug Abuse, we conducted a survey to characterize the epidemiology of cannabidiol use between October 25, 2023 and November 3, 2023 to collect data on CBD use behaviors among US adults. Respondents were recruited from a national online panel of approximately 55,000 panelists maintained by the market research company Ipsos Public Affairs (formerly GfK Group). This panel is called KnowledgePanel® and is the largest probability-based online panel in the US, representing 97% of the adult population (31). It uses address-based stratified random sampling to recruit adult households, including those with only cell phones and no landline phones. To increase representativeness, households that lack access to the internet are provided with free internet services and/or devices to enable their participation and interviews can be conducted in both English or Spanish. The KnowledgePanel® has been widely used to provide nationally representative statistics on drug use (32–40) and produces estimates that have higher external validity than nonprobability based sampling approaches (41).

For this study, a random sample of 4,505 panel members was drawn from KnowledgePanel®, 2,880 of whom responded to the invitation, completed the screening questionnaire, and provided written consent to participate. After a detailed description of CBD and its distinct differences from tetrahydrocannabinol (THC), the screening questionnaire asked if respondents had ever used CBD in their lifetime. A predetermined target sample of *n* = 1,000 ever CBD users and n = 500 non-users of CBD was selected to provide representative estimates of CBD use behaviors with a ± 3% margin of error. In total, *n* = 1,523 qualified for the survey (*n* = 1,008 CBD ever users and *n* = 515 never users of CBD). This yielded a final-stage completion rate of 63.9% (2,880/4,505) and a qualification rate of 53.0% (1,523/2,880). The survey was completed in a median of 10 min. The Human Research Protections Program at the University of California San Diego considered this survey exempt human subjects research. The survey was in accordance with the STROBE checklist for cross-sectional studies (42).

### Survey questions

#### Ever and past-year CBD use

Respondents were asked, “Have you ever used a CBD product before, even once?” Those who responded “yes” were considered to be ever users of CBD. Ever users were then asked “Have you used a CBD product in the past 12 months, even once?” and those who responded “yes” were considered past-year users.

#### Substitute CBD use

CBD users were asked, “Have you ever stopped using a medication and replaced it with a CBD product?” Those who responded “yes” were considered to use CBD as a substitute and then asked, “What medication(s) did you stop taking and replace with a CBD product?” and provided up to 10 open-text fields to record medications. For every listed medication, respondents were asked, “What was the main health condition you were taking this medication for?” and responded in an open-text field.

#### Adjunct CBD use

Similarly, CBD users were also asked, “Have you ever used a CBD product at the same time as another medication to treat the same medical condition?” Those who responded “yes” were considered to use CBD as an adjunct and then asked, “What medication(s) did you use in addition to CBD?” and provided up to 10 open-text fields to record medications. For every listed medication, respondents were asked, “What was the main health condition you were taking this medication for?” and responded in an open-text field.

#### Reasons for using CBD as a substitute or adjunct

Respondents who reported using CBD as a substitute or adjunct to another medication were asked to describe their reasons in an open-ended prompt. For example, substitute users were asked: “In the space provided below, can you briefly describe your motivation for stopping the use of a medication and replacing it with CBD? Please write at least 2–3 sentences.” Adjunct users were asked a parallel question about their motivation for using CBD at the same time as another medication to treat the same condition. Responses were collected as free text and later coded into thematic categories for analysis.

#### CBD ever use frequency

Ever CBD users were asked, “How many times have you used CBD products in your entire life?” Responses were categorized as 1 time, 2 to 10 times, 11 to 20 times, 21 to 50 times, 51 to 99 times, or 100 + times.

#### Past-year CBD use days

Ever CBD users were asked, “Approximately how many days in total in the past 12 months did you use CBD products?” Responses were categorized as 0 days, 1 day, 2 to 30 days, 31 to 99 days, or 100 + days.

#### Typical CBD dose (past 12 months)

Ever CBD users were asked, “Can you please provide your best estimate of how many milligrams you usually took per day on the days you used CBD in the past 12 months?” Responses were categorized as 1 to 10 milligrams (mg), 11 to 25 mg, 26 to 50 mg, 51 to 100 mg, 101 to 500 mg, 501 to 1000 mg, 1000 + mg, do not know, or did not use in the past 12 months.

#### Mode of consumption used most often

Ever CBD users were asked, “In your lifetime, which type of CBD product have you ever used, even once? Check all that apply,” with modes including: joints (dry CBD-dominant “hemp” flower rolled in paper); pipes/bowls/bongs (filtration devices used to smoke CBD-dominant “hemp” flower); concentrate vaping (electronic vaporizers used to heat CBD concentrates such as wax/hash oil/budder/shatter at low temperatures to inhale vapor); dry-flower vaping (electronic vaporizers used to heat CBD-dominant “hemp” flower at low temperatures to inhale vapor); dabbing (heating CBD concentrates to very high temperatures on a hot surface to inhale vapor); edibles (CBD baked or infused into foods/snacks/candy); drinks (beverages infused with CBD or mixed with CBD concentrates); tinctures or oils (liquid CBD extracts ingested orally or taken under the tongue); sprays (oral sprays dispensed under the tongue or inside the cheek); pills/tablets/capsules (oral CBD formulations); patches (transdermal patches that release CBD through the skin); and topicals (lotions/salves/bath salts/oils applied to the skin, including cosmetics and sex lubricants). Respondents who selected two or more lifetime modes were then asked, “In your lifetime, which type of CBD product have you used most often?” If respondents selected only one lifetime mode, that mode was auto-assigned as the “most often” mode.

#### CBD product type used most often

Ever CBD users were asked which CBD product types they had ever used (check all that apply) and then were asked, “In your lifetime, which type of CBD product have you used most often?” If respondents reported only one lifetime product type, that type was auto-assigned as the “most often” type. Product types were defined as: full-spectrum (contains all natural cannabis/hemp compounds including THC, with THC ≤ 0.3%), broad-spectrum (contains multiple cannabis/hemp compounds with all or most THC removed; trace THC may remain), and CBD isolate (contains only CBD and should contain 0% THC). Respondents could also report “do not know”.

#### Clinician recommendation for CBD use

Ever CBD users were asked, “Has any of your CBD use ever been recommended by a doctor or other health professionals?” Response options were: Yes, ALL of my CBD use; Yes, PART of my CBD use; or No, NONE of my CBD use.

#### CBD-attributed adverse events

Ever CBD users were asked, “Have you ever, even once, had a health problem that you believe resulted from your CBD use?” Those who responded “yes” were asked to describe the health problem(s) in an open-text field. Do not know/refused responses to the open-text item were treated as missing (i.e., excluded from contributing to the adverse-event numerator). In addition, during coding we removed cases where symptoms were ambiguous or where the respondent did not describe symptoms.

### Coding medical conditions

Medical conditions reported by individuals who used CBD products as adjunct or substitute and symptoms listed in CBD-attributed adverse events were coded using the Medical Dictionary for Regulatory Activities (MedDRA) (27.1). MedDRA is a dictionary of medical terminology developed under the auspices of the International Conference on Harmonization of Technical Requirements for Registration of Pharmaceuticals for Human Use. Regulatory authorities widely use MedDRA, including to track medical reasons for product use in the FDA Adverse Event Reporting System (43). MedDRA uses a five-level hierarchical structure with the “System Organ Class” (SOC) as the broadest classification tier. SOC represents terms based on an anatomical or physiological system, etiology, or function. Three levels beneath the SOC is the “Preferred Term” (PT), which represents a distinct medical concept related to a symptom, sign, or disease diagnosis. The lowest tier in the hierarchy is the “Lowest-Level Term” (LLT), which captures how observations are described in everyday language by a consumer. One example of a hierarchy is “joint pain” as the LLT followed by “arthralgia” as the PT and “musculoskeletal and connective tissue disorders” as the SOC. Throughout this manuscript, the term ‘disorder’ is used according to MedDRA’s standardized nomenclature. This does not necessarily indicate a formal diagnosis under the International Classification of Diseases or the Diagnostic and Statistical Manual of Mental Disorders.

To translate medical conditions to MedDRA, six annotators (N. M. R., P. L., G. A., S. E. E., J. B., and L. B.) were assigned in pairs to independently code all medical conditions to MedDRA LLTs. Annotators had moderate agreement on LLT selections (Cohen’s K = 0.69), with differences primarily resulting from lexical variations allowed in MedDRA (e.g., MedDRA has distinct LLTs for “difficulty sleeping” and “poor sleep”). Coder agreement was higher at the PT (Cohen’s K = 0.88) and SOC (Cohen’s K = 0.90) levels, therefore analyses were performed at these levels. Disagreements were resolved by unanimous agreement between two study staff (E. A. C. A. and E. C. L.).

### Coding medications

The medications reported by individuals who used CBD were coded using RxNav English Language Version 2.9.177 available from the National Institutes of Health’s National Library of Medicine. RxNav assigns a Concept Unique Identifier (RxCUI) to each individual drug entity which can be used to identify and distinguish it from other drugs. These RxCUIs were then linked back to the RxNorm Name of each drug. To translate medications to RxNav, six annotators (N. M. R., P. L., G. A., S. E. E., J. B., and L. B.) were assigned in pairs to independently code all medications to a RxCUI by extracting a medication name from the original respondent text and searching for it in the RxNAV web dashboard (44). Medications listed in RxNav were recorded to the RxNav term. Medications not in RxNav, were assigned a general category (e.g., “anxiety pills” would be coded as “anxiety medications”). There was high agreement between annotators on medication coding (Cohen’s K = 0.85).

### Thematic analysis of reasons for using CBD as a substitute or adjunct

Two authors (E. A. C. A. and L. B.) reviewed all open-text responses using an inductive thematic approach (45). First, these authors read responses in full and developed a set of six thematic codes reflecting distinct motivations for using CBD as a substitute or adjunct: (1) experimentation (trying CBD to see if it provided additional relief), (2) preference for natural remedies, (3) side effect avoidance, (4) dependence avoidance, (5) medical recommendation, and (6) lack of access to medication. Then E. A. C. A. and L. B. re-read all responses and independently assigned one or more thematic labels to each response (no restriction on the number of themes per response). Interrater reliability was assessed on a shared subset of responses (*n* = 297). Agreement was high across themes (percent agreement range: 88.9–99.3%), with substantial to near-perfect concordance (Cohen’s *κ* range: 0.66–0.94 across individual themes; κ = 0.67 for the overall combined 6-bit pattern). All disagreements were adjudicated by E. A. C. A., and the adjudicated codes were used in final analyses.

### Statistical analyses

To produce population-based estimates, we used survey weights constructed by Ipsos, which accounted for oversampling of CBD users in the study design (see eMethods for additional information on how weights were constructed). All percentages were weighted by population parameters based on the findings of the March 2023 supplement of the US Census Bureau’s Current Population Survey. A survey-specific post-stratification adjustment was used to account for survey non-response, as well as non-coverage or undersampling and oversampling resulting from the survey-specific sampling design. Weighted percentages and the corresponding 95% confidence intervals (CIs) were calculated for ever and past-year CBD use among all US adults. Among ever CBD users, we estimated weighted distributions of self-reported CBD use characteristics, including lifetime frequency, number of days used in the past 12 months, usual dose on days used in the past 12 months, mode of consumption used most often, CBD product type used most often, clinician recommendation, and self-reported CBD-attributed adverse events. Among respondents who reported using CBD as a substitute and/or adjunct, we estimated the weighted prevalence of each thematic reason for substitute/adjunct use. Among CBD users, we also summarized self-reported medical conditions and medications for which CBD was used as a substitute or adjunct treatment. All analyses were performed using R, version 4.1 (R Project for Statistical Computing).

## Results

**Figure 1:**
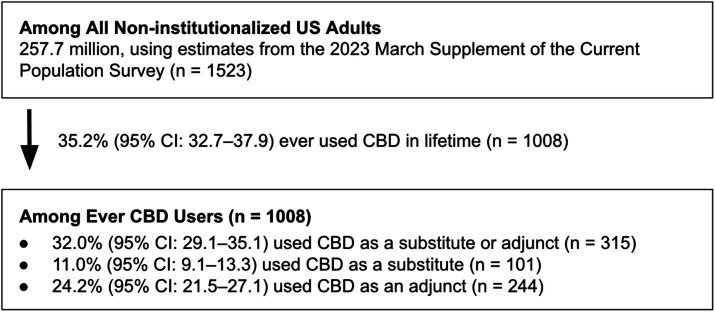
Estimates of CBD use in the US adult population. US, United States; CBD, Cannabidiol; CI, Confidence interval.

As of October/November 2023, an estimated 35.2% (95% CI: 32.7–37.9) of US adults reported ever using CBD in their lifetime, representing 90.8 million US adults and 21.8% (95% CI: 20.0–23.8) of US adults reported using CBD in the past 12 months (Figure 1). Of these ever CBD users, 32.0% (95% CI: 29.1–35.1) reported using CBD as either a substitute or adjunct. Among ever CBD users, use of CBD as an adjunct (24.2%; 95% CI: 21.5–27.1) was more prevalent than use as a substitute (11.0%; 95% CI: 9.1–13.3), and 3.3% (95% CI: 2.3–4.8) reported using CBD as both a substitute and adjunct at some point in their lifetime.

In Table 1, we summarize self-reported characteristics of CBD use among ever CBD users (*n* = 1008). Most users reported relatively infrequent ever use, with 39.6% (95% CI: 36.4–42.9) reporting use 2 to 10 times and 16.6% (95% CI: 14.2–19.2) reporting 11 to 20 times; however, 15.0% (95% CI: 12.7–17.5) reported use 100 or more times. In the past 12 months, 38.7% (95% CI: 35.5–41.9) reported 0 days of use, while 33.1% (95% CI: 30.1–36.2) reported 2 to 30 days, 13.6% (95% CI: 11.5–16.0) reported 31 to 99 days, and 9.0% (95% CI: 7.3–11.1) reported 100 + days. Most respondents did not know the typical CBD dose they took on days they used CBD in the past 12 months (19.3%; 95% CI: 16.9–22.0). Among those reporting a dose, 15.6% (95% CI: 13.4–18.1) reported typically taking 1 to 10 mg per day of use. The most commonly reported primary modes of consumption were topicals (26.0%; 95% CI: 23.3–28.9), edibles (e.g., gummies) (25.2%; 95% CI: 22.5–28.2), tinctures/oils (14.6%; 95% CI: 12.5–17.0), and joints (13.1%; 95% CI: 11.0–15.6). Among ever CBD users, 20.6% (95% CI: 18.1–23.3) reported primarily using full-spectrum products, 14.4% (95% CI: 12.3–16.8) reported broad-spectrum products, and 14.9% (95% CI: 12.8–17.3) reported CBD isolate; however, half of users did not know the type of CBD product they used most often (50.1%; 95% CI: 46.9–53.3). Most respondents reported that none of their CBD use had been recommended by a doctor or other health professional (82.3%; 95% CI: 79.6–84.7). A small proportion reported ever having a health problem they believed resulted from CBD use (2.4%; 95% CI: 1.5–3.6), and most symptoms appeared to be related to accidental exposure to THC (e.g., anxiety, euphoric mood, heart rate increased, or increased need for sleep).

**Table 1:** Self-reported characteristics of use among US adult cannabidiol users (*n* = 1008).

Characteristics of CBD use	Percent of ever CBD users, % (95% CI)
The number of times CBD was used in entire lifetime	
1 time	8.7% (7.0–10.8)
2 to 10 times	39.6% (36.4–42.9)
11 to 20 times	16.6% (14.2–19.2)
21 to 50 times	14.2% (12.0–16.7)
51 to 99 times	5.9% (4.5–7.6)
100 or more times	15.0% (12.7–17.5)
Approximate number of days of CBD use in the past 12 months	
0 days	38.7% (35.5–41.9)
1 day	5.6% (4.3–7.4)
2 to 30 days	33.1% (30.1–36.2)
31 to 99 days	13.6% (11.5–16.0)
100 + days	9.0% (7.3–11.1)
Milligrams usually taken per day on the days you used CBD in the past 12 months	
1 to 10 mg	15.6% (13.4–18.1)
11 to 25 mg	8.8% (7.1–10.8)
26 to 50 mg	6.3% (4.9–8.1)
51 to 100 mg	4.8% (3.6–6.5)
101 to 500 mg	3.9% (2.8–5.4)
501–1000 mg	2.0% (1.3–3.2)
More than 1000 mg	1.6% (0.9–2.6)
Do not know	19.3% (16.9–22.0)
Did not use in the past 12 months	37.6% (34.6–40.8)
Mode of consuming CBD products used most often	
Joints	13.1% (11.0–15.6)
Pipes, bowls, or bongs	5.0% (3.8–6.7)
Concentrate Vaping	7.5% (5.8–9.5)
Dry Flower Vaping	1.2% (0.6–2.2)
Dabbing	1.3% (0.7–2.4)
Edibles (e.g., gummy)	25.2% (22.5–28.2)
Drinks	1.3% (0.7–2.1)
Tinctures or oils	14.6% (12.5–17.0)
Sprays	0.7% (0.3–1.7)
Pills or capsules	3.7% (2.6–5.0)
Patch	0.4% (0.2–1.2)
Topicals	26.0% (23.3–28.9)
Type of CBD product used most often^a^	
Full-Spectrum	20.6% (18.1–23.3)
Broad-Spectrum	14.4% (12.3–16.8)
Isolate	14.9% (12.8–17.3)
Do not know	50.1% (46.9–53.3)
Has any of your CBD use ever been recommended by a doctor or other health professionals?	
All of it	6.9% (5.4–8.8)
Part of it	10.8% (8.9–13.0)
None of it	82.3% (79.6–84.7)
Have you ever, even once, had a health problem that you believe resulted from your CBD use?^b^	
Yes	2.4% (1.5–3.6)
No	97.6% (96.4–98.5)

US, United States; CBD, Cannabidiol; CI, Confidence interval; mg, milligram(s); THC, Tetrahydrocannabinol. ^a^Full-spectrum: Contains the plant’s full range of cannabinoids/compounds, including THC, with THC limited to ≤0.3%; Broad-spectrum: Contains multiple cannabis/hemp compounds, but THC is removed entirely or nearly entirely; trace THC may remain; CBD isolate: Contains CBD only; the most purified form and should contain 0% THC. ^b^Symptoms described in adverse events: Abdominal discomfort; Anxiety; Diarrhea; Dizziness; Euphoric mood; Headache; Heart rate increased; Increased need for sleep; Lethargy; Loss of consciousness; Malaise; Nausea; Pain; Postural orthostatic tachycardia syndrome; Psychotic disorder; Renal failure; Renal pain; Syncope.

Of the SOCs indicated in the use of CBD as a substitute or adjunct, musculoskeletal and connective tissue disorders were the most common, cited by 10.1% of ever CBD users (95% CI: 8.4–12.2) (Table 2). Following that were psychiatric disorders (7.4%; 95% CI: 5.9–9.2), general disorders and administration site conditions (e.g., pain) (6.8%; 95% CI: 5.4–8.6), nervous system disorders (2.9%; 95% CI: 2.0–4.2), and surgical and medical procedures (1.0%; 95% CI: 0.5–1.9). All other categories were cited by fewer than 1% of ever CBD users.

**Table 2:** Self-reported uses of cannabidiol as a substitute or adjunct among US adult cannabidiol users by MedDRA system organ class related MedDRA preferred terms (*n* = 1008).

MedDRA system organ class	Related MedDRA preferred terms	Percent of ever CBD users, % (95% CI)
Musculoskeletal and connective tissue disorders	Arthralgia, Arthritis, Arthropathy, Back pain, Fibromyalgia, Intervertebral disc degeneration, Joint stiffness, Muscle spasms, Muscle tightness, Myalgia, Myositis, Neck pain, Osteoarthritis, Osteoporosis, Pain in extremity, Plantar fasciitis, Psoriatic arthropathy, Rheumatoid arthritis, Rotator cuff syndrome, Scoliosis, Spinal osteoarthritis, Tendonitis	10.1% (8.4–12.2)
Psychiatric disorders	Anxiety, Attention deficit hyperactivity disorder, Bipolar disorder, Bipolar I disorder, Borderline personality disorder, Depression, Generalized anxiety disorder, Insomnia, Mental disorder, Nervousness, Panic attack, Panic reaction, Post-traumatic stress disorder, Sleep disorder, Stress	7.4% (5.9–9.2)
General disorders and administration site conditions	Feeling of relaxation, General physical health deterioration, Ill-defined disorder, Inflammation, Pain, Procedural pain	6.8% (5.4–8.6)
Nervous system disorders	Carpal tunnel syndrome, Epilepsy, Headache, Hypoaesthesia, Migraine, Multiple sclerosis, Neuralgia, Neuropathy peripheral, Paraesthesia, Parkinson’s disease, Sciatica, Seizure	2.9% (2.0–4.2)
Surgical and medical procedures	Foot operation, Hip arthroplasty, Joint arthroplasty, Knee arthroplasty, Knee operation, Pain management, Shoulder operation, Surgery, Vulvectomy	1.0% (0.5–1.9)

US, United States; MedDRA, Medical Dictionary for Regulatory Activities; CI, Confidence interval; HIV, Human immunodeficiency virus; CBD, Cannabidiol. ^a^Values <1% (alphabetized by MedDRA System Organ Class then by related MedDRA Preferred Terms): Cardiac disorders (Cardiomyopathy, Postural orthostatic tachycardia syndrome); Congenital, familial and genetic disorders (Sickle cell disease); Ear and labyrinth disorders (Vertigo); Gastrointestinal disorders (Colitis, Colitis ulcerative, Crohn’s disease, Dyspepsia, Gastrooesophageal reflux disease, Toothache); Immune system disorders (Multiple allergies); Infections and infestations (Herpes zoster, HIV infection, Sinusitis, Tooth infection); Injury, poisoning and procedural complications (Ankle fracture, Back injury, Injury, Ligament rupture, Limb injury, Muscle injury, Nerve injury); Investigations (Blood cholesterol, Blood pressure abnormal, Blood pressure measurement, Cortisol abnormal); Metabolism and nutrition disorders (Diabetes mellitus, Glucose tolerance impaired, Gout); Neoplasms benign, malignant and unspecified (incl cysts and polyps) (Breast cancer); Reproductive system and breast disorders (Erectile dysfunction); Respiratory, thoracic and mediastinal disorders (Asthma, Dyspnoea, Sleep apnoea syndrome); Vascular disorders (Hypertension).

In Table 3, we list all 99 unique PTs indicated in the use of CBD as a substitute or adjunct. The PTs reported by at least 1% of ever CBD users included pain (5.5, 95% CI: 4.3–7.2), anxiety (4.9, 95% CI: 3.7–6.4), back pain (3.1, 95% CI: 2.2–4.4), arthralgia (e.g., joint pain) (3.0, 95% CI: 2.1–4.3), arthritis (2.7, 95% CI: 1.9–3.8), depression (2.2, 95% CI: 1.5–3.4), and myalgia (e.g., muscle pain) (1.0, 95% CI: 0.5–1.8).

**Table 3:** Self-reported uses of cannabidiol as a substitute or adjunct among US adult cannabidiol users by MedDRA preferred terms (*n* = 1008).

Related MedDRA preferred terms	Percent of ever CBD users, % (95% CI)
Pain	5.5% (4.3–7.2)
Anxiety	4.9% (3.7–6.4)
Back pain	3.1% (2.2–4.4)
Arthralgia	3.0% (2.1–4.3)
Arthritis	2.7% (1.9–3.8)
Depression	2.2% (1.5–3.4)
Myalgia	1.0% (0.5–1.8)

US, United States; MedDRA, Medical Dictionary for Regulatory Activities; CI, Confidence interval; HIV, Human immunodeficiency virus; CBD, Cannabidiol. ^a^Values <1% (alphabetized): Ankle fracture, Arthropathy, Asthma, Attention deficit hyperactivity disorder, Back injury, Bipolar disorder, Bipolar I disorder, Blood cholesterol, Blood pressure abnormal, Blood pressure measurement, Breast cancer, Cardiomyopathy, Carpal tunnel syndrome, Colitis, Colitis ulcerative, Cortisol abnormal, Crohn’s disease, Diabetes mellitus, Dyspepsia, Dyspnoea, Epilepsy, Erectile dysfunction, Feeling of relaxation, Fibromyalgia, Foot operation, Gastrooesophageal reflux disease, Generalized anxiety disorder, General physical health deterioration, Glucose tolerance impaired, Gout, Headache, Herpes zoster, Hip arthroplasty, HIV infection, Hypertension, Hypoaesthesia, Ill-defined disorder, Inflammation, Injury, Insomnia, Intervertebral disc degeneration, Joint arthroplasty, Joint stiffness, Knee arthroplasty, Knee operation, Ligament rupture, Limb injury, Mental disorder, Migraine, Multiple allergies, Multiple sclerosis, Muscle injury, Muscle spasms, Muscle tightness, Myositis, Neck pain, Nerve injury, Neuralgia, Nervousness, Neuropathy peripheral, Osteoarthritis, Osteoporosis, Pain in extremity, Pain management, Panic attack, Panic reaction, Paraesthesia, Parkinson’s disease, Plantar fasciitis, Post-traumatic stress disorder, Postural orthostatic tachycardia syndrome, Procedural pain, Psoriatic arthropathy, Rheumatoid arthritis, Rotator cuff syndrome, Sciatica, Scoliosis, Seizure, Shoulder operation, Sickle cell disease, Sinusitis, Sleep apnoea syndrome, Sleep disorder, Spinal osteoarthritis, Stress, Surgery, Tendonitis, Tooth infection, Toothache, Vertigo, Vulvectomy.

In Table 4, we list 139 unique medications for which CBD was used as a substitute or adjunct treatment. The medications that were substituted or used as an adjunct with CBD by at least 1% of ever CBD users were ibuprofen (4.8, 95% CI: 3.6–6.3), Tylenol (3.9, 95% CI: 2.8–5.3), pain medications (no brand or generic listed) (2.9, 95% CI: 2.0–4.2), gabapentin (1.4, 95% CI: 0.8–2.3), Advil (1.2, 95% CI: 0.7–2.1), and anxiety medications (no brand or generic listed) (1.1, 95% CI: 0.6–2.1).

**Table 4:** Medications substituted or used as an adjunct with cannabidiol by RxNav term and related MedDRA preferred terms among US adult cannabidiol users (*n* = 1008).

RxNav medication terms	Percent of ever CBD users, % (95% CI)
Ibuprofen	4.8% (3.6–6.3)
Tylenol	3.9% (2.8–5.3)
Pain medications^a^	2.9% (2.0–4.2)
Gabapentin	1.4% (0.8–2.3)
Advil	1.2% (0.7–2.1)
Anxiety medications^a^	1.1% (0.6–2.1)

MedDRA, Medical Dictionary for Regulatory Activities; US, United States; CI, Confidence interval; CBD, Cannabidiol. ^a^Medications not found in RxNav are labeled by a general category. ᵇValues <1% (alphabetized): Abilify, Absorbine veterinary liniment, Acetaminophen, Acetaminophen / phenylephrine, Adderall, Albuterol, Aleve, Amantadine, Ambien, Antibiotic medicationsᵃ, Arthritis medicationsᵃ, Aspercreme, Aspirin, Asthma medicationsᵃ, Ativan, Aubagio, Avsola, Azathioprine, Azithromycin, Bayer aspirin pill, Benadryl, Bengay, Biofreeze, Blood pressure medicationsᵃ, Bupropion, Buspar, Buspirone, Cannabis, Capsaicin, Carbidopa / levodopa, Celebrex, Celexa, Cimzia, Citalopram, Cymbalta, Depakote, Depression medicationsᵃ, Diclofenac, Dietary supplements, Diazepam, Divalproex, Diphenhydramine, Duloxetine, Elbow brace, Enbrel, Flexeril, Fluoxetine, Herbal medicationsᵃ, Hydralazine, Hydrocodone, Hydrocortisone, Hydroxychloroquine, Hydroxyzine, Hydroxyzine pamoate, Icy hot, Imitrex, Indomethacin, Inflammation medicationsᵃ, Insulin, Klonopin, Lamotrigine, Lexapro, Lidocaine, Lipator, Lipitor, Lisinopril, Lithium, Lorazepam, Lunesta, Maxalt, Meclizine, Melatonin, Meloxicam, Mesalamine, Metformin, Methocarbamol, Metoprolol, Midodrine, Midol, Migraine medicationsᵃ, Marinol, Morphine, Motrin, Muscle relaxer medicationsᵃ, Narcotic medicationsᵃ, Naropin, Naprosyn, Naproxen, Nausea medicationsᵃ, Nerve medicationsᵃ, Non-steroidal anti-inflammatory drugsᵃ, Norco, Nortriptyline, Nurtec, Odefsey, Omeprazole, Orphenadrine, Oxycodone, Oxycontin, Parkinson’s medicationsᵃ, Paxil, Pepcid, Percocet, Post traumatic stress disorder medicationsᵃ, Pramipexole, Prednisone, Pregabalin, Psychosis medicationsᵃ, Seizure medicationsᵃ, Sertraline, Seroquel, Simvastatin, Sleep medicationsᵃ, Stelara, Stress medicationsᵃ, Suboxone, Sulfasalazine, Symbicort, Tizanidine, Topamax, Topical product, Traumeel, Tramadol, Trintellix, Vicodin, Viagra, Voltaren, Wellbutrin, Xanax, Zoloft, Zyloprim.

In the supplement, we report the prevalence of each medication specific to the use of CBD as a substitute (Supplementary eTable 1) and adjunct (Supplementary eTable 2). The medications that were substituted by at least 1% of CBD users were pain medications (no brand or generic listed) (1.4%; 95% CI: 0.8–2.5), ibuprofen (1.2%; 95% CI: 0.7–2.2), and anxiety medications (no brand or generic listed) (1.0%; 95% CI: 0.5–1.9). The medications that were used as an adjunct with CBD by at least 1% of CBD users were ibuprofen (3.8%; 95% CI: 2.8–5.2), Tylenol (3.0%; 95% CI: 2.1–4.3), pain medications (no brand or generic listed) (1.5%; 95% CI: 0.9–2.5), gabapentin (1.3%; 95% CI: 0.8–2.2), and Advil (1.0%; 95% CI: 0.6–1.9).

In Table 5, we summarize themes from open-coded free-text responses describing why CBD was used as a substitute or adjunct to medications among respondents who reported substitute and/or adjunct use (*n* = 315). Most respondents described their use of CBD as an adjunct or substitute as merely experimentation (84.1%; 95% CI: 74.5–90.5), often noting they wanted to see whether CBD provided additional relief or a calming effect. Other motivations included preference for “natural” remedies (20.7%; 95% CI: 13.2–30.8), avoiding medication side effects (16.9%; 95% CI: 10.3–26.4), and avoiding dependence or addiction risk (16.9%; 95% CI: 10.5–26.3). A minority of respondents cited medical recommendation (6.5%; 95% CI: 3.0–13.3) or lack of access to other medications typically involving pain applications (5.4%; 95% CI: 2.0–13.9).

**Table 5:** Reasons given for why cannabidiol was substituted or used as an adjunct to another medications among, among US adults who used CBD as a substitute or adjunct (*n* = 315).

Theme^a^	Examples^b^	Percent of ever CBD users who used as either a substitute or adjunct, % (95% CI)
Experimentation	“I wanted to find out if it would give me additional relief. I wanted the calming effect”.	84.1% (74.5–90.5)
Preference for natural remedies	“CBD is natural, non addictive and keeps money away from big pharma”.	20.7% (13.2–30.8)
Side effect avoidance	“The medications I was prescribed were causing too many side effects”.	16.9% (10.3–26.4)
Dependence avoidance	“Oxycodone was not relieving the pain. I did not want to take additional doses or the same dose for additional days because of fear of overdose or addiction”.	16.9% (10.5–26.3)
Medical recommendation	“My doctor recommended trying it to reduce pain”.	6.5% (3.0–13.3)
Lack of access to medication	“It was not my choice to stop taking the medicine, it was the government telling doctors to stop giving the medicine to people due to the drug problem. But people like me and my Husband and people we know with chronic pain, those medicines helped so we had to find alternative ways to get just a little bit of pain relief and CBD and THC is the only way to get rid of the pain for just a little bit”.	5.4% (2.0–13.9)

Because responses could be assigned multiple themes, percentages do not sum to 100%. US, United States; CBD, Cannabidiol; CI, Confidence interval; THC, Tetrahydrocannabinol. ^a^Themes were developed through open-coding of free-text responses. See methods for details. ^b^Responses were edited slightly to improve clarity.

## Discussion

In this nationally representative survey conducted in October–November 2023, 35.2% of US adults reported ever using CBD and 21.8% reported 12 month use. The past 12 month estimate closely aligns with a 21.1% prevalence found in a June 2023 nationally representative survey (30), and is about one percentage point higher than the 20.6% estimate from the National Survey on Drug Use and Health in 2022 (29). Nearly one-third of ever CBD users in our sample reported using CBD as either a substitute or adjunct for conventional therapies, most often for pain and anxiety. Although some of these applications (e.g., pain management) are being explored in clinical trials with promising results (46, 47), no FDA-approved CBD treatments currently exist for these indications, indicating a substantial divergence between consumer use and regulatory guidelines.

A key finding is the widespread use of CBD in combination with prescription or over-the-counter medications, highlighting a potential for adverse drug–drug interactions. CBD can inhibit or induce cytochrome P450 (CYP450) enzymes, thereby altering the metabolism of numerous drugs, including anticoagulants (e.g., warfarin), antiepileptics (e.g., clobazam), antidepressants (e.g., fluoxetine), and sedatives (e.g., diazepam) (48, 49). Elevated serum concentrations may intensify adverse effects, such as bleeding with warfarin (50), while induction of CYP450 could hasten the breakdown of certain drugs (e.g., carbamazepine), diminishing their efficacy (48, 51). High CBD doses can also lead to liver enzyme elevations, potentially signaling liver injury (7, 11, 51). One study of healthy volunteers receiving 1,500 mg of CBD daily found that about half exhibited elevated liver enzymes, and approximately 6% had levels that may be clinically concerning (52). Concerns also arise when CBD is combined with over-the-counter analgesics or prescription agents such as acetaminophen (Tylenol) and gabapentin, which can contribute to hepatic injury or altered drug metabolism, respectively (11, 48). Animal models suggest that doses comparable to 700 mg CBD and 2,800 mg acetaminophen in a 70 kg adult may interact to cause severe liver damage, although direct human evidence remains limited (50).

However, it is important to consider that much of the published evidence on cannabidiol drug interactions and hepatic enzyme effects cited above comes from clinical or experimental settings that use relatively high daily doses (including ~1500 mg/day) and/or standardized pharmaceutical formulations, which may not reflect typical use patterns in the general population. In our sample, very few CBD users (1.6%) reported using products containing more than 1000 mg total CBD and most reported using CBD infrequently, suggesting that the highest-dose scenarios emphasized in prior studies likely apply to a small subset of users. Accordingly, our discussion of pharmacologic interactions is intended to highlight biologically plausible risks—particularly for people using higher-dose products, using CBD frequently, or taking narrow-therapeutic-index medications—rather than to imply that clinically meaningful interactions are common among all CBD users. Indeed, self-reported adverse events were rare (2.4%) and this is generally consistent with evidence suggesting that CBD is well tolerated (53–55). Nonetheless, a requirement for “Drug Facts” warnings on CBD products could help consumers identify possible contraindications, especially if they take anticoagulants, psychotropics, antiepileptics, immunosuppressants, or drink alcohol frequently.

In considering the risk/benefit of consumers potentially substituting CBD for approved medications, while stopping a treatment with some known benefit in favor of CBD where the benefit is unproved presents some risk, it needs also to be acknowledged that commonly prescribed medicines, e.g., psychotropics for anxiety or opioids for pain, also have potentially severe adverse effects and risk for forming. To the extent that CBD, which has very few side effects and likely very low risk of dependence at low to moderate doses, helps a consumer reduce anxiety and decrease or discontinue a medication with more side effects or risk for forming dependence, this might be viewed as a benefit.

Drug–drug interactions also should not be interpreted only as a source of harm. The same pharmacologic mechanisms that warrant caution—e.g., CBD’s capacity to alter drug exposure through CYP450 enzymes (49, 56)—also create a plausible opportunity to learn when co-administration could be clinically useful. In principle, this could include identifying combinations where CBD helps achieve comparable symptom control with lower doses of certain medications, potentially reducing dose-related adverse effects, versus combinations where it meaningfully increases risk (e.g., toxicity, oversedation, or diminished efficacy). For example, given that many respondents described using CBD specifically as an adjunct or substitute for chronic pain, a research agenda that evaluates both risk mitigation and therapeutic optimization would match real-world use and could yield more actionable clinical guidance for such common endpoints.

In our survey, 37.6% of CBD users could not report how much CBD was in the product they used, and 50.1% did not know whether the product contained THC. This uncertainty is consequential because several reported adverse events—such as dizziness, anxiety/panic symptoms, and euphoria—are consistent with unintentional exposure to THC or other psychotropic cannabinoids. Although self-reported symptoms cannot establish causation and these effects may also reflect underlying conditions or concomitant medications, the pattern underscores a practical gap in consumer-facing product information. Prior work has documented that CBD products are frequently mislabeled with respect to cannabinoid content and that inadequate disclosure of CBD/THC amounts can create risks of unintended THC exposure (57). More recent analytic testing likewise suggests substantial real-world deviations between labeled and measured CBD potency and frequent misclassification of “isolate/broad-spectrum/full-spectrum” claims—further reinforcing that consumers may be unable to accurately infer dose or THC exposure from packaging alone (58). When users cannot reliably identify dose or THC content, they cannot meaningfully titrate use, anticipate impairment, or avoid interactions. A policy approach that prioritizes clear, standardized labeling (e.g., total mg CBD per package and per serving, THC presence and amount, and prominent intoxication/impairment warnings), coupled with basic quality assurance to reduce mislabeling and inadvertent exposure to intoxicating constituents, could help consumers make safer, more informed decisions.

### Limitations

This study’s cross-sectional design provides a snapshot of CBD use in October–November 2023 and limits interpretation in several important ways. Because exposures and outcomes were assessed at the same time, we cannot infer causality or directionality. For example we cannot determine whether CBD use preceded changes in medication use, whether symptoms or side effects motivated substitution/adjunct use, or whether any reported CBD-attributed health problems were caused by CBD rather than underlying conditions or co-administered therapies. Similarly, we cannot evaluate temporal trends (e.g., whether substitution/adjunct use is increasing or decreasing) or within-person changes in patterns of use over time. In addition, the survey did not capture respondents’ underlying medical history in a way that would support clinically meaningful stratification (e.g., chronic pain cohorts or respondents with psychiatric diagnoses); therefore, we were unable to conduct sensitivity analyses across these patient subgroups, and heterogeneity in substitution/adjunct use by clinical context may be obscured. Additional limitations include reliance on self-report (with potential recall error and misclassification, including vague medication names), likely underestimation of concomitant medication use because adjunct use was restricted to co-administration for the same condition, and the absence of detailed information on the CBD product being used as a substitute or adjunct or use of multiple products. Although we analyzed an item on CBD product type (isolate, broad-spectrum, or full-spectrum), we did not collect quantitative information on THC content (including THC-to-CBD ratios) or co-use of THC. Consistent with our focus on CBD-dominant products, we therefore could not directly compare potential risks and perceived benefits against products with higher THC content—an important direction for future research given that many of the same therapeutic endpoints (e.g., chronic pain, sleep, anxiety) motivate use of both CBD- and THC-containing products. Finally, results are limited to adults and to CBD specifically and do not address other loosely regulated cannabinoid products that may pose similar or greater risks and be used for their perceived therapeutic benefit (59).

## Conclusion

These findings highlight that millions of US adults use CBD as a substitute and adjunct for a wide range of health conditions—most of which lack FDA-approved indications or clear evidence-based guidance. At present, FDA approval for prescription CBD remains limited to a narrow set of seizure disorders, which should not be interpreted as proof of no therapeutic potential for other endpoints, but rather as reflecting in part the difficulty of generating regulatory-grade evidence in a landscape where cannabis has long been federally controlled and clinical research has faced substantial structural barriers (60, 61). Recent federal actions underscore this point. In December 2025, the White House explicitly framed the gap between widespread medical use and limited clinical evidence as leaving patients and clinicians without adequate guidance, and directed agencies to reduce barriers and expand medical marijuana and CBD research, which hopefully will allow for rigorous clinical evaluation of CBD and medical cannabis for promising endpoints (62). In the interim, clinical care should emphasize open, nonjudgmental communication so that patients feel comfortable disclosing CBD use, alongside a pragmatic, harm-reduction approach focused on safety. This includes reviewing concomitant medications, discussing uncertainty in dose and product composition (including possible THC exposure), monitoring for adverse effects in higher-risk situations (e.g., individuals using higher doses), and helping patients identify more reliable products and appropriate follow-up when symptoms emerge. While available clinical evidence generally suggests CBD is well tolerated (53–55), stronger evidence on efficacy, dosing, modes of consumption, product quality, and clinically meaningful interaction management is still needed so that widespread real-world use is better supported by clear guidance and translates into safer, more effective care.

## Data Availability

The original contributions presented in the study are included in the article/Supplementary material, further inquiries can be directed to the corresponding author.
