# Supplementary material for: Self-reported use of cannabidiol as a substitute or adjunct for approved medications
Source: Front Public Health. 2026 Feb 6;14:1720348. doi: 10.3389/fpubh.2026.1720348 (PMC12920505; doi:10.3389/fpubh.2026.1720348)
Supplement: Supplementary file 1 [file Data_Sheet_1.pdf]

Supplemental Online Content

eMethods. Additional information on Ipsos KnowledgePanel Weighting.

eTable 1: Medications substituted with cannabidiol by RxNav term among US adult cannabidiol users (n=1008)

eTable 2: Medications used as an adjunct with cannabidiol by RxNav term among US adult cannabidiol users (n=1008)

This supplemental material has been provided by the authors to give readers additional information about their work.

eMethods. Additional information on Ipsos KnowledgePanel Weighting

Once all survey data have been collected and processed, design weights are adjusted to account for any differential nonresponse that may have occurred. Depending on the specific target population for a given study, geodemographic distributions for the corresponding population are obtained from the CPS, the U.S. Census Bureau's American Community Survey (ACS), or in certain instances from the weighted KnowledgePanel profile data. For this purpose, an iterative proportional fitting (raking) procedure is used to produce the final weights. In the final step, calculated weights are examined to identify and, if necessary, trim outliers at the extreme upper and lower tails of the weight distribution. The resulting weights are then scaled to aggregate to the total sample size of all eligible respondents.

For this study, our weighting process included the following steps:

Step 1: Design weights for KnowledgePanel (KP) assignees were computed to reflect their modeled selection probabilities.

Step 2: The above design weights for all screened respondents, include those who refused or answered "don't know" the ever CBD product usage screening question (CBD_EVR_01), were weighted to represent the following geodemographic distributions of the 18 and over US population using an iterative proportional fitting (raking) procedure. The needed benchmarks were obtained from the 2023 March Supplement of the Current Population Survey (CPS), except language dominance within Hispanics, which is not available from CPS, were obtained from the 2021 American Community Survey (ACS).

- Gender (Male, Female) by Age (18-29, 30-44, 45-59, 60+)
- Race-Ethnicity (White/Non-Hispanic, Black/Non-Hispanic, Other/Non-Hispanic, Hispanic,
- 2+ Races/Non-Hispanic)
- Census Region (Northeast, Midwest, South, West) by Metropolitan Status (Metro, Non-
- Metro)
- Education (Less than High School, High School, Some College, Bachelor or higher)
- Household Income (under $25K, $25K-$49,999, $50K-$74,999, $75K-$99,999, $100K-
- $149,999, $150K and over)
- Language Dominance (English Dominant Hispanic, Bilingual Hispanic, Spanish Dominant
- Hispanic, Non-Hispanic)

The weights were scaled to add up to the number of screened respondents (named as screen_wt).

Step 3: All screened respondents who have and have not ever used CBD products were separated out and benchmarks for the respective population were created using their screener weight (screen_wt). These benchmarks were used in weighting the final qualified respondents.

Step 4: Screener weights (screen_wt) for final qualified respondents were raked to the following geodemographic distributions of the 18 and over population who have and have not ever used CBD products, such that final qualified respondents within the two subgroups (CBD ever users and non-CBD ever users) were weighted to be representative of their respective population and the two subgroups were weighted in their proper proportion.

- Gender (Male, Female) by Age (18-29, 30-44, 45-59, 60+) by Ever Used CBD Products
- (Yes, No)
- Race-Ethnicity (White/Non-Hispanic, Black/Non-Hispanic, Other/Non-Hispanic, Hispanic,
- 2+ Races/Non-Hispanic) by Ever Used CBD Products (Yes, No) --- collapsed Other and 2+
- Races in Non-CBD user
- Census Region (Northeast, Midwest, South, West) by Ever Used CBD Products (Yes, No)
- Metropolitan Status (Metro, Non-Metro) by Ever Used CBD Products (Yes, No)
- Education (Less than High School, High School, Some College, Bachelor or higher) by
- Ever Used CBD Products (Yes, No)
- Household Income (under $25K, $25K-$49,999, $50K-$74,999, $75K-$99,999, $100K-
- $149,999, $150K and over) by Ever Used CBD Products (Yes, No)
- Language Dominance (English Dominant Hispanic, Bilingual/Spanish Dominant Hispanic, Non-Hispanic) by Ever Used CBD Products (Yes, No)

The resulting weights were trimmed and scaled to add up to the number of qualified respondents (named as qualify_wt).

eTable 1: Medications substituted with cannabidiol by RxNav term among US adult cannabidiol users (n=1008)

RxNav Medication Terms	Percent of lifetime CBD users, % (95% CI)
Pain medications[a]	1.4% (0.8-2.5)
Ibuprofen	1.2% (0.7-2.2)
Anxiety medications[a]	1.0% (0.5-1.9)
Tylenol	0.8% (0.4-1.7)
Depression medications[a]	0.5% (0.2-1.3)
Topical product	0.5% (0.2-1.1)
Aspirin	0.4% (0.2-1.0)
Hydrocodone	0.4% (0.1-1.0)
Xanax	0.3% (0.1-1.1)
Oxycontin	0.3% (0.1-1.3)
Zoloft	0.3% (0.1-1.1)
Motrin	0.3% (0.1-1.0)
Melatonin	0.3% (0.1-1.1)
Icy hot	0.3% (0.1-0.9)
Sleep medications[a]	0.3% (0.1-0.9)
Percocet	0.2% (0.1-0.9)
Advil	0.2% (0.0-0.9)
Acetaminophen	0.2% (0.0-0.7)
Oxycodone	0.2% (0.0-0.7)
Naproxen	0.2% (0.0-1.1)
Orphenadrine	0.2% (0.0-1.1)
Lithium	0.2% (0.0-1.1)
Diazepam	0.1% (0.0-1.0)
Midol	0.1% (0.0-1.0)
Duloxetine	0.1% (0.0-1.0)
Metformin	0.1% (0.0-0.9)
Psychosis medications[a]	0.1% (0.0-0.9)
Imitrex	0.1% (0.0-0.9)
Celexa	0.1% (0.0-0.9)
Klonopin	0.1% (0.0-0.9)
Lisinopril	0.1% (0.0-0.9)

Seizure medications[a]	0.1% (0.0-0.9)
Bengay	0.1% (0.0-0.9)
Narcotic medications[a]	0.1% (0.0-0.8)
Blood pressure medications[a]	0.1% (0.0-0.8)
Marinol	0.1% (0.0-0.8)
Norco	0.1% (0.0-0.7)
Omeprazole	0.1% (0.0-0.7)
Seroquel	0.1% (0.0-0.7)
Sertraline	0.1% (0.0-0.7)
Topamax	0.1% (0.0-0.7)
Acetaminophen / phenylephrine	0.1% (0.0-0.7)
Gabapentin	0.1% (0.0-0.7)
Ambien	0.1% (0.0-0.7)
Meclizine	0.1% (0.0-0.7)
Nerve medications[a]	0.1% (0.0-0.7)
Lunesta	0.1% (0.0-0.6)
Depakote	0.1% (0.0-0.6)
Buspirone	0.1% (0.0-0.6)
Non-steroidal anti-inflammatory drugs[a]	0.1% (0.0-0.6)
Wellbutrin	0.1% (0.0-0.6)
Lexapro	0.1% (0.0-0.6)
Voltaren	0.1% (0.0-0.6)
Morphine	0.1% (0.0-0.5)
Vicodin	0.1% (0.0-0.5)
Aspercreme	0.1% (0.0-0.5)
Meloxicam	0.1% (0.0-0.5)
Ativan	0.1% (0.0-0.5)
Parkinson's medications[a]	0.1% (0.0-0.5)
Muscle relaxer medications[a]	0.1% (0.0-0.4)
Arthritis medications[a]	0.0% (0.0-0.3)

[a]Medications not found in RxNav are labeled by a General Category

eTable 2: Medications used as an adjunct with cannabidiol by RxNav term among US adult cannabidiol users (n=1008)

RxNav Medication Terms	Percent of lifetime CBD users, % (95% CI)
Ibuprofen	3.8% (2.8-5.2)
Tylenol	3.0% (2.1-4.3)
Pain medications[a]	1.5% (0.9-2.5)
Gabapentin	1.3% (0.8-2.2)
Advil	1.0% (0.6-1.9)
Acetaminophen	0.8% (0.4-1.6)
Cannabis	0.6% (0.3-1.3)
Hydrocodone	0.4% (0.2-1.1)
Anxiety medications[a]	0.4% (0.1-1.2)
Xanax	0.4% (0.1-1.0)
Lexapro	0.4% (0.1-1.0)
Wellbutrin	0.4% (0.1-1.1)
Aleve	0.4% (0.2-0.9)
Meloxicam	0.4% (0.1-0.9)
Aspirin	0.3% (0.1-0.9)
Flexeril	0.3% (0.1-0.9)
Zoloft	0.3% (0.1-1.1)
Melatonin	0.3% (0.1-0.9)
Celexa	0.3% (0.1-1.0)
Naproxen	0.3% (0.1-0.9)
Oxycodone	0.3% (0.1-0.8)
Sertraline	0.3% (0.1-0.9)
Naprosyn	0.3% (0.1-1.0)
Buspirone	0.3% (0.1-0.9)
Tramadol	0.3% (0.1-0.8)
Topical product	0.3% (0.1-0.9)
Depression medications[a]	0.3% (0.1-0.9)
Blood pressure medications[a]	0.3% (0.1-0.9)
Hydroxychloroquine	0.3% (0.1-0.8)
Citalopram	0.3% (0.1-0.8)
Fluoxetine	0.2% (0.1-1.0)

Percocet	0.2% (0.1-0.9)
Post traumatic stress disorder medications[a]	0.2% (0.1-0.9)
Hydroxyzine	0.2% (0.1-0.9)
Sleep medications[a]	0.2% (0.0-0.9)
Pepcid	0.2% (0.1-0.8)
Lorazepam	0.2% (0.0-0.8)
Inflammation medications[a]	0.2% (0.0-0.8)
Simvastatin	0.2% (0.0-0.7)
Aspercreme	0.2% (0.0-0.8)
Antibiotic medications[a]	0.2% (0.0-1.3)
Cymbalta	0.2% (0.0-0.7)
Diclofenac	0.2% (0.0-0.7)
Azithromycin	0.2% (0.0-1.2)
Psychosis medications[a]	0.2% (0.0-1.2)
Tizanidine	0.2% (0.0-0.7)
Duloxetine	0.2% (0.0-0.7)
Depakote	0.2% (0.0-0.7)
Arthritis medications[a]	0.2% (0.0-0.6)
Voltaren	0.2% (0.0-0.6)
Muscle relaxer medications[a]	0.1% (0.0-0.6)
Pregabalin	0.1% (0.0-0.6)
Azathioprine	0.1% (0.0-0.6)
Klonopin	0.1% (0.0-0.9)
Lisinopril	0.1% (0.0-0.9)
Viagra	0.1% (0.0-0.9)
Suboxone	0.1% (0.0-0.9)
Naropin	0.1% (0.0-0.9)
Metoprolol	0.1% (0.0-0.5)
Asthma medications[a]	0.1% (0.0-0.8)
Aubagio	0.1% (0.0-0.8)
Lipator	0.1% (0.0-0.8)
Lidocaine	0.1% (0.0-0.8)
Benadryl	0.1% (0.0-0.8)
Stress medications[a]	0.1% (0.0-0.7)
Ambien	0.1% (0.0-0.7)

Diphenhydramine	0.1% (0.0-0.7)
Herbal medications[a]	0.1% (0.0-0.7)
Insulin	0.1% (0.0-0.7)
Symbicort	0.1% (0.0-0.7)
Oxytocin	0.1% (0.0-0.7)
Humira	0.1% (0.0-0.7)
Stelara	0.1% (0.0-0.7)
Hydrocortisone	0.1% (0.0-0.7)
Midodrine	0.1% (0.0-0.7)
Indomethacin	0.1% (0.0-0.7)
Biofreeze	0.1% (0.0-0.7)
Bayer aspirin pill	0.1% (0.0-0.7)
Motrin	0.1% (0.0-0.7)
Icy hot	0.1% (0.0-0.7)
Bupropion	0.1% (0.0-0.7)
Celebrex	0.1% (0.0-0.7)
Traumeel	0.1% (0.0-0.6)
Odefsey	0.1% (0.0-0.6)
Absorbine veterinary liniment	0.1% (0.0-0.6)
Amantadine	0.1% (0.0-0.6)
Carbidopa / levodopa	0.1% (0.0-0.6)
Pramipexole	0.1% (0.0-0.6)
Nausea medications[a]	0.1% (0.0-0.6)
Zyloprim	0.1% (0.0-0.6)
Topamax	0.1% (0.0-0.6)
Mesalamine	0.1% (0.0-0.6)
Paxil	0.1% (0.0-0.6)
Capsaicin	0.1% (0.0-0.6)
Elbow brace	0.1% (0.0-0.6)
Lipitor	0.1% (0.0-0.6)
Divalproex	0.1% (0.0-0.6)
Cimzia	0.1% (0.0-0.6)
Methocarbamol	0.1% (0.0-0.6)
Oxycontin	0.1% (0.0-0.6)
Non-steroidal anti-inflammatory drugs[a]	0.1% (0.0-0.6)

Buspar	0.1% (0.0-0.6)
Adderall	0.1% (0.0-0.5)
Norco	0.1% (0.0-0.5)
Prednisone	0.1% (0.0-0.5)
Sulfasalazine	0.1% (0.0-0.5)
Maxalt	0.1% (0.0-0.5)
Nortriptyline	0.1% (0.0-0.5)
Nurtec	0.1% (0.0-0.4)
Vicodin	0.1% (0.0-0.4)
Enbrel	0.1% (0.0-0.4)
Avsola	0.1% (0.0-0.4)
Dietary supplements	0.1% (0.0-0.4)
Albuterol	0.0% (0.0-0.3)
Hydralazine	0.0% (0.0-0.3)
Abilify	0.0% (0.0-0.3)
Trintellix	0.0% (0.0-0.3)
Migraine medications[a]	0.0% (0.0-0.3)
Hydroxyzine pamoate	0.0% (0.0-0.3)
Lamotrigine	0.0% (0.0-0.3)

[a]Medications not found in RxNav are labeled by a General Category
